# An Envenoming Syndrome from Massive *Vespa* Stings Induces Multiple Organ Failure

**DOI:** 10.3390/insects11040219

**Published:** 2020-04-02

**Authors:** Tse-Hao Chen, Wan-Ting Liao, Chien-Sheng Chen, Po-Chen Lin, Meng-Yu Wu

**Affiliations:** 1 Department of Emergency Medicine, MacKay Memorial Hospital, Taipei 104, Taiwan; joechenid@gmail.com; 2 Institute of Medicine, Chung Shan Medical University, Taichung 402, Taiwan; enolainsky@gmail.com; 3 Chinese Medicine Department, Show Chwan Memorial Hospital, Changhua 500, Taiwan; 4 Department of Emergency Medicine, Taipei Tzu Chi Hospital, Buddhist Tzu Chi Medical Foundation, New Taipei 231, Taiwan; holeyeye@yahoo.com.tw (C.-S.C.); taipeitzuchier@gmail.com (P.-C.L.); 5 Department of Emergency Medicine, School of Medicine, Tzu Chi University, Hualien 970, Taiwan

**Keywords:** bee, multiple organ failure, acute kidney injury, rhabdomyolysis, acute liver failure

## Abstract

Envenoming syndrome is a systemic reaction induced by inoculation of large volumes of Hymenoptera venom. The clinical manifestations range from skin allergic reactions to multiple organ failure. Vespid venom-induced toxic reactions and anaphylaxis are the most common lethal mechanism of death, involving acute respiratory failure, acute liver failure, rhabdomyolysis, acute kidney injury, and severe coagulopathy. Multiple organ failure as a consequence of severe venom toxicity is a rare but dangerous complication in victims. Delay of intervention to correct vespid venom-induced toxic reactions may cause catastrophic complications. Here, we describe a case presenting a rare vespid venom-induced multiple organ failure with systemic coagulopathy after massive *Vespa* attack.

## 1. Introduction

Fatal envenoming syndrome is a rare clinical entity, which is characterized by a systemic reaction induced by inoculation of large volumes of Hymenoptera venom. Vespid sting victims may present with an anaphylactic reaction, which should be detected and managed initially to prevent a fatal situation. Initially, a skin reaction is usually detected and accompanied by local tenderness. Massive envenomation is defined as a victim who has suffered from more than 50 stings in a single event. The patient may present with anaphylactic shock [1]. The typical symptoms include tachycardia, dyspnea, and consciousness loss. Patients with massive envenomation also develop the potential life-threatening triad of acute kidney injury (AKI), intravascular hemolysis, and rhabdomyolysis [2]. Timely recognition of envenoming syndrome and early correction of anaphylactic shock are very important for emergency physicians to break the vicious circle of fatal envenoming syndrome. Here, we describe a rare case presenting with rhabdomyolysis, AKI, intravascular hemolysis, and acute respiratory distress syndrome (ARDS) due to massive envenomation with multiple organ failure. The etiology and clinical features of *Vespa*-induced envenoming syndrome are discussed.

## 2. Case Presentation

**Figure 1:**
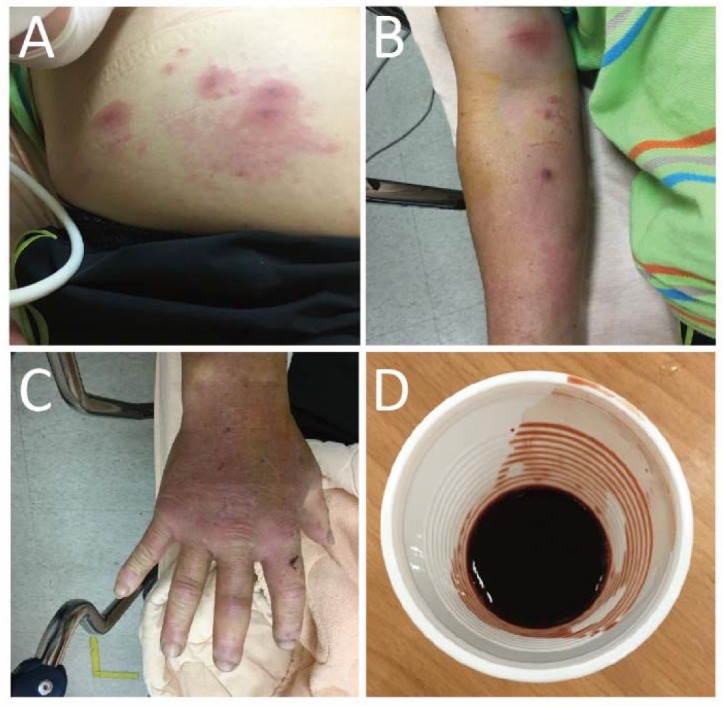
(**A**–**C**) An erythematous rash with central necrosis and local tenderness was noted over the face, abdomen, and extremities. (**D**) Acute gross hematuria occurred after admission.

**Figure 2:**
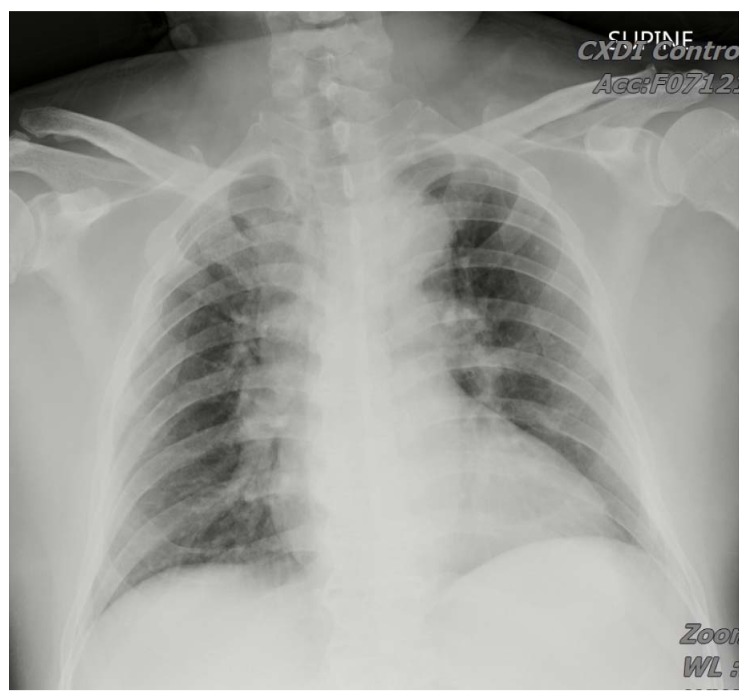
The chest X-ray revealed bilateral diffuse infiltration.

**Figure 3:**
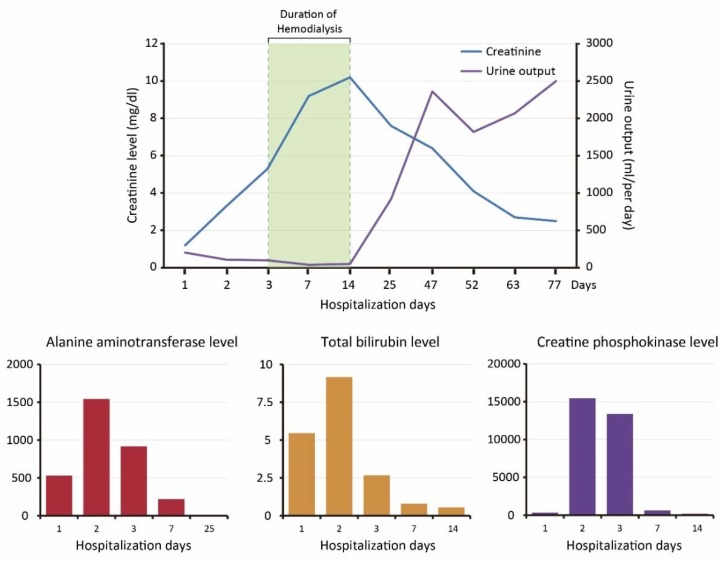
The follow-up laboratory data progression in a clinical case of multiple organ failure.

A 67-year-old male presented to the emergency department due to multiple *Vespa* stings (>50 stings) 30 min ago. He had no past medical history or medicine history. During transfer to our hospital, a severe allergic reaction was suspected due to massive *Vespa* stings with tachycardia and tachypnea. Epinephrine 0.3 mg intramuscular injection was administered to prevent anaphylactic shock before arrival of an emergency medical technician. A generalized erythematous rash over the face, abdomen, and extremities, causing a painful sensation, was noted (Figure 1A–C). On physical examination, his temperature was 35.4 °C, blood pressure was 109/74 mmHg, heart rate was 103 beats/min, and Glasgow Coma Scale (GCS) score was E_4_V_5_M_6_. The erythematous rash and sting wounds were noted over the face, abdomen, and extremities (Figure 1A–C). The sting wounds initially showed mild cutaneous central hemorrhage at the sting site, surrounding an edematous and reddish inflammatory area. The cutaneous central hemorrhage at sting site progressed to dark brown necrotic tissue with local pain. The ulcerated lesions were painful and produced local heat. Bilateral breath sound was clear without wheezing or crackle. Intramuscular (IM) injections of diphenhydramine 30 mg, and dexamethasone 4 mg were given initially for anaphylactic reaction after being stung. Gross hematuria occurred after one hour (Figure 1D). To rule out Takotsubo cardiomyopathy or Kounis syndrome, we also studied ECG and cardiac enzyme data. The SpO2 was up to 99–100% under room temperature conditions. The ECG showed normal sinus rhythm without ST segment elevation or depression. The series follow-up cardiac enzyme test showed no significant elevation. In ICU, the follow-up ECG monitoring showed no significant sign of Kounis syndrome. There was no evidence of Takotsubo cardiomyopathy in bedside echo. The laboratory evaluation in the patient revealed severe acute hepatitis, AKI, coagulopathy, and rhabdomyolysis (Table 1). Patient experienced shortness of breath and facial swelling after 12 h of hospital stay. Emergency epinephrine 1 mg inhalation and 0.5 mg IM injection were used to control airway edema but, unfortunately, progressive dyspnea and stridor with desaturation occurred. During emergency intubation, swelling of vocal cords was noticed. Chest radiography showed normal heart size with bilateral lung diffused infiltration, which was compatible with ARDS (Figure 2). Ventilation support was conducted consecutively. Metabolic acidosis with anuria encountered after hospitalization. We administered furosemide and sodium bicarbonate as forced alkaline diuresis to prevent further AKI progression. However, progressive kidney failure with hyperkalemia occurred (Cre: 9.2 mg/dL; K: 5.5 mmol/L); he underwent hemodialysis three days after admission. Hemodialysis was ceased on hospital stay day 15, owing to adequate urine output and much improved creatinine levels (Cre 2.5 mg/dL) (Figure 3). He received hemodialysis a total of five times. Plasma infusion was applied intermittently. Coagulopathy and hepatitis resolved on hospital stay day 42. Because of difficulty weaning from mechanical ventilation, the patient underwent tracheostomy tube insertion on day 44 and it was removed on day 77. No dyspnea or desaturation occurred afterwards. He was discharged on day 90 in a stable condition.

## 3. Discussion

Stinging accidents with vespid venom are a notable cause of anaphylactic shock [3]. Several components have been identified in vespid venom, including histamine, phospholipase A, Antigen-5, kinin, melittin, phospholipase, and hyaluronidase. Histamine is one major component that induces an anaphylactic reaction. Melittin, a membrane-active polypeptide, may cause release of inflammatory and pro-inflammatory mediators via degranulation of mast cells. It also has pain-producing effects in the nervous system [4]. Phospholipase A may trigger immunoglobulin E (IgE)-mediated allergic reactions via formation of membrane pores leading to cellular lysis and hemolysis. Other protein enzymes, such as phospholipase and hyaluronidase, are involved in systemic allergic reactions. In people with few stings, the allergic reaction caused by direct toxicity of venom may be restrained at the site of the sting. Massive *Vespa* stings may lead to a systemic reaction, including serum sickness and multiple organ dysfunction syndrome, with a high mortality rate [5]. In the acute phase, hypersensitivity reactions are directly caused by massive envenomation or mediated by immunoglobulin E (IgE) antibodies. In the delay phase, serum sickness may occur after three days of envenomation with immune-complex related syndromes, such as disseminated intravascular coagulation and thrombocytopenic purpura [6]. The severity of vespid venom-induced toxic reactions is usually based on the number of stings in a single event. Sampson’s severity score has been used to classify patients into five grades to help pre-hospital emergency medical technicians to identify the need for epinephrine [7]. Conditions in which epinephrine injection is recommended include the sensation of throat pruritus, difficulty swallowing, dyspnea, dysrhythmia, hypotension, and loss of consciousness. Early administration of epinephrine should be considered in patients with a Sampson’s severity score of more than grade 3 [3]. 

Several complications of massive *Vespa* stings were found in our case, including AKI, rhabdomyolysis, hepatitis, coagulopathy, and ARDS. AKI may be induced by two major pathways. After massive envenomation, the phospholipases (PLAs), histamine, and hyaluronidase may cause AKI via rhabdomyolysis and hemolysis, which is compatible with a high creatine-phosphokinase (CPK) value, and aggressive coagulopathy with prolonged prothrombin time (PT), and activated partial thromboplastin time (APTT) in laboratory evaluation. In addition, venom toxicity directly damaged renal tubules, causing a nephrotoxic effect [8]. Tahura and Hanif [9] reported 18 cases of children with AKI after multiple *Vespa* stings revealed acute tubulointerstitial nephritis in renal pathology. The anaphylactic shock caused by PLAs and Antigen-5 from multiple *Vespa* stings may also lead to acute interstitial nephritis. In our patient, gross hematuria accompanied with elevated indirect bilirubin value (total bilirubin: 5.45 mg/dL; direct bilirubin: 0.24 mg/dL) also indicated that the patient encountered an episode of hemolysis. After aggressive fluid supplementation and timely hemodialysis, the oliguric AKI was corrected with recovery of renal function [8]. The direct toxicity of massive envenomation also promoted hepatitis [8,10]. Interestingly, venom toxicity that developed into ARDS has been rarely reported previously. The exact mechanism as to why ARDS was triggered remained unclear. The release of excessive inflammatory cytokines and complementary cascade activation may be involved [11]. Our patient developed ARDS in a late anaphylactic phase. Ventilator support was applied to protect the lungs. However, our patient suffered from difficult extubation due to several complications of massive envenomation. Tracheostomy tube insertion was conducted temporarily and was finally removed before discharge. In addition, the multiple skin lesions were improved from necrotic lesion to scar. In Youichi Yanagawa et al.’s [12] report, skin lesions presented with cutaneous hemorrhage at wasp sting site; then, cutaneous hemorrhage changed into a necrotic lesion after 24 h. In our patient, the same skin lesions presented with cutaneous hemorrhage and some lesions were necrotic lesions surrounded by an edematous and reddish inflammatory area. After one week, the central necrotic lesions became scars without local tenderness.

Envenoming syndrome and anaphylaxis reaction both occurred in our patient. The early administration of epinephrine controlled the progression of the anaphylaxis reaction. Rhabdomyolysis, coagulopathy, and acute kidney injury are typical presentations of envenoming syndrome in cases of massive Vespa stings. Coagulopathy may lead to hypovolemic shock. Therefore, early, adequate fluid resuscitation can help prevent shock status. Acute renal failure may have resulted from direct kidney injury by direct toxicity of venom or secondarily from rhabdomyolysis. In addition, shock or disseminated intravascular coagulation may induce acute tubular necrosis, which is another mechanism of acute kidney injury. In our patient, bleeding disorder was noted but there was no significant sign of shock after timely resuscitation. The increased creatine-kinase and creatinine levels were compatible with rhabdomyolysis and acute kidney injury. Timely hemodialysis improved the renal function and electrolyte imbalance.

Early diagnosis along with an appropriate therapeutic strategy are important in cases of vespid venom-induced envenoming syndrome. In emergency departments, immediate control of anaphylaxis and shock status are critical principles. Sting sites should also be closely observed for possible infectious signs rather than just allergic reactions. For local reactions, oral analgesics, including NSAIDs, and antihistamines, may limit symptoms. A corticosteroid should be considered for large local reactions. For an anaphylactic reaction, administration of IM epinephrine 0.3 to 0.5 mg or EpiPens may help early control of anaphylactic shock. Venom immunotherapy (VIT) is a potential treatment for sting reactions. VIT may reduce subsequent systemic sting reactions with efficacy reported to be 84–96% for Hymenoptera venom [13,14]. Based on the 2017 The European Academy of Allergy and Clinical Immunology (EAACI) guidelines, VIT should be considered in patients with grade II anaphylactic reactions [15]. We highlight how early intervention in cases of allergic reaction and anaphylactic shock assists recovery by discussing a rare case of vespid venom-induced envenoming syndrome which presented several complications. This syndrome should be kept in mind in patients with Vespa stings because early management of anaphylaxis can prevent catastrophic complications by recognizing envenoming syndrome in clinical prognosis.

## 4. Conclusions

Vespid venom-induced envenoming syndrome is the most common lethal mechanism of death in massive *Vespa* attack, involving acute respiratory failure, acute liver failure, rhabdomyolysis, acute kidney injury, and severe coagulopathy. Multiple organ failure as a consequence of severe venom toxicity is a rare but dangerous complication in victims. Early intervention to correct vespid venom-induced toxic reactions may prevent catastrophic complications.

## Figures and Tables

**Table 1:** The laboratory evaluation of this patient.

Variables	Normal Range	Patient Data						
		Day 1	Day 2	Day 3	Day 4	Day 7	Day 8	Day 10
White cell count	3.9–10.6 (× 10^3^/µL)	17.42	23.07	21.02	---	20.45	---	18.54
Hemoglobin	13.5–17.5 g/dL	17.0	15.8	12.0	---	9.0	---	7.7
Platelet counts	150–400 (× 10^3^/µL)	299	241	132	---	104	---	190
Band	0–3%	0.0	10.0	5.0	---	5.0	---	0.0
Monocyte	2–10%	5.0	7.0	3.0	---	9.0	---	7.0
Neutrophile	40–75%	54.0	73.0	87.0	---	80.0	---	80.0
Lymphocyte	20–45%	39.0	9.0	4.0	---	4.0	---	8.0
Eosinophile	1–6%	0.0	0.0	0.0	---	0.0	---	2.0
PT	8–12 sec	127.2	14.8	12.3	---	---	---	---
APTT	23.9–35.5 sec	58.1	67.6	35.4	---	---	---	---
INR	----	14.51	1.48	1.22	---	---	---	---
FDP-D-dimer	0–500 µg/L	857.92	---	---	---	---	---	---
Fibrinogen	200-400 mg/dL	218.9	---	---	---	---	---	---
Creatinine	0.7–1.3 mg/dL	1.2	3.3	5.3	5.9	9.2	---	8.3
Sodium	136–145 mmole/L	135	131	131	136	136	---	141
Potassium	3.5–5.1 mmole/L	3.7	4.8	4.4	4.5	5.4	---	4.1
LDH	85-227 IU/L	2318	---	---	---	---	---	---
Glucose	3.9–5.6 mmole/L	158	---	---	---	---	---	---
ALT	16–63 U/L	531	1541	917	542	221	---	110
AST	15–37 U/L	1782	---	2034	374	49	---	25
Creatine kinase	39–308 IU/L	324	15,439	12,210	6218	619	---	179
CKMB	7–25 IU/L	149	313	---	---	---	---	---
Total bilirubin	0.0–1.0 mg/dL	5.48	9.15	2.67	1.70	0.79	---	1.01
Direct bilirubin	0.0–0.3 mg/dL	0.24	---	0.88	---	---	---	---
Myoglobin	17.4–105.7 ng/mL	----	>3893	>3893	---	---	---	---
pH	7.35–7.45	----	----	----	---	---	7.49	---
pCO_2_	35–45 mmHg	----	----	----	---	---	29.3	---
pO_2_	80–100 mmHg	----	----	----	---	---	218.9	---
HCO_3_	22–26 mmole/L	----	---	----	---	---	22.0	---

PT: prothrombin time; APTT: activated partial thromboplastin time; ALT: alanine aminotransferase; AST: aspartate aminotransferase.
